# Organoids: Key advances, optimization, and technological iterations in their application to neurodegenerative diseases

**DOI:** 10.4103/NRR.NRR-D-25-00924

**Published:** 2025-12-30

**Authors:** Jiangyu Zhao, Jing Wang, Xing Guo

**Affiliations:** Department of Neurobiology, School of Basic Medical Sciences, Nanjing Medical University, Nanjing, Jiangsu Province, China

**Keywords:** Alzheimer disease, amyotrophic lateral sclerosis, bioprinting, frontotemporal dementia, Huntington’s disease, microfluidics, neurodegenerative diseases, organoids, Parkinson’s disease, transplantation

## Abstract

Organoid technology, as an innovative approach, has shown great potential in disease modeling, target screening, and the development of treatment strategies. However, traditional organoids still have three major limitations in research: the absence of specific cell types, the lack of blood–brain barrier structure, and insufficient reproducibility of experimental results. In recent years, researchers have gradually overcome these limitations by introducing innovative techniques such as advanced culture methods, microfluidic systems, bioprinting, organoid transplantation, and assembloid construction. This progress has facilitated the widespread application of organoids in the study of neurodegenerative diseases. This paper aims to systematically review the technological innovations of organoids in the study of neurodegenerative diseases. By summarizing classical organoid construction strategies and their limitations, it emphasizes the value of organoids in comprehensive applications within neurodegenerative disease research. In this review, we focus on five specific neurodegenerative diseases: Alzheimer’s disease, Parkinson’s disease, Huntington’s disease, amyotrophic lateral sclerosis, and frontotemporal dementia. Research in these diseases demonstrates that organoids improve experimental accessibility and reduce development cycles in disease modeling, target discovery, and therapeutic strategy formation. Using customized equipment and gene editing techniques, these organoids can be tailored to specific needs, providing pathophysiologically relevant disease models and enhancing our understanding of neurodegenerative diseases. Although organoid technology has demonstrated significant advantages in disease research, its potential for treating neurodegenerative diseases has not yet been fully explored, which may become an important direction for future research.

**Facts**

• Simulation of 3D structures and complex microenvironments: Organ-on-a-chip technology successfully simulates the complex microenvironment of the brain through 3D culture and microfluidic techniques, including the neural vascular unit and inter-regional interactions. This innovation allows organ-on-a-chip models to more accurately reflect the physiological state of the human brain.

• Diversity of cell types and functions: By utilizing gene editing and customized culture methods, researchers have successfully introduced multiple cell types (such as microglia and vascular endothelial cells) into organ-on-a-chip models, achieving functional neural networks. This addresses the limitation of traditional models, which typically feature a narrow range of cell types.

• Portability and enhanced functionality: Organ-on-a-chip models can be integrated with host tissues through transplantation techniques, resulting in improved vascularization and functional support. This enhances the longevity of the culture and increases the physiological relevance of the models.

**Open questions**

• How can the standardization and large-scale production of complex multi-regional organoids be achieved to meet the high reproducibility and high-throughput requirements necessary for efficient drug screening and personalized medicine?

• What methods can be used to simulate more complex and precise biological structures to elucidate the interactions between different regions in neurodevelopmental disorders?

• What are the most significant ethical and regulatory obstacles in advancing organoid therapies (including transplantation and organoid-derived products) from preclinical validation in neurodevelopmental disorders to clinical trials?

## Introduction

Neurodegenerative diseases (NDDs) are a group of disorders characterized by ongoing degeneration and dysfunction of neurons (Agnello and Ciaccio, 2022). These debilitating conditions, including Alzheimer’s disease (AD), Parkinson’s disease (PD), Huntington’s disease (HD), amyotrophic lateral sclerosis (ALS), and frontotemporal dementia (FTD), remarkably impact patients’ lives and pose a considerable public health challenge (Festa et al., 2024; Livingston et al., 2024; Zimmer et al., 2024). The complex nature of their development, which involves interactions among genetic, environmental, and molecular factors, has hindered progress in developing effective treatments (Ben-Shlomo et al., 2024). Researchers have traditionally focused primarily on animal models and cell cultures. Although these models provide valuable insights, they often fail to accurately replicate the actual disease scenario (Creekmore et al., 2024). Therefore, developing an accurate and efficient experimental model is an urgent priority.

Originating from groundbreaking innovations in stem cell technology, the emergence of organoid technology has opened new avenues for studying NDDs. These three-dimensional (3D) structures represent a pioneering frontier in exploring human brain development and pathology (Coronel et al., 2026). As a novel tool for examining diverse sets of human cell types interacting within a 3D framework, organoids provide improved access to the etiopathogenesis of NDDs (Xu et al., 2023). Early organoids featured non-vascularized and oligocellular environments, which limited their ability to replicate the *in vivo* cytoarchitecture necessary for modeling NDDs (Kim and Chang, 2023). Technological innovations, including advanced culture methods (Qian et al., 2018; Patel et al., 2024), microfluidic systems (Abdulla et al., 2023), bioprinting (Juraski et al., 2023), transplantation of organoids (Wang et al., 2023), and assembloids (Kim et al., 2024a), have addressed these challenges.

**Figure 1:**
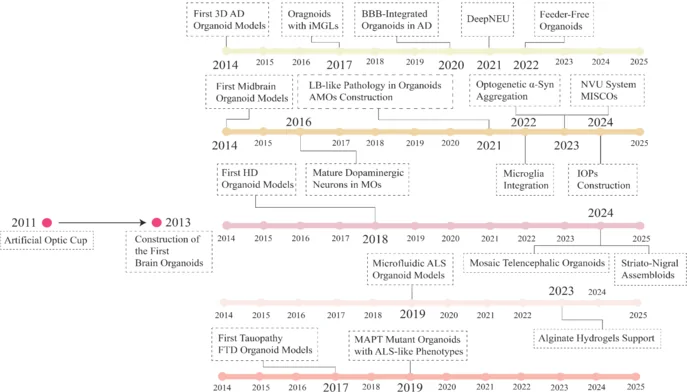
Timeline of the advancements of organoids in neurodegenerative disease research. AD: Alzheimer’s disease; ALS: amyotrophic lateral sclerosis; AMOs: automated midbrain organoids; BBB: blood–brain barrier; FTD: frontotemporal dementia; HD: Huntington’s disease; iMGLs: iPSC-derived microglia-like cells; IOPs: inter organoid projections; LB: Lewy body: MAPT: microtubule-associated protein tau; MISCOs: ventral midbrain-striatum-cortex assembloids; MOs: midbrain organoids; NVU: neurovascular unit.

This review summarizes the traditional construction methods of organoids and highlights some of their limitations. We believe that recent technological innovations can address many of these issues. We emphasize that these optimized organoids can be practically used in research on specific NDDs, aiding all aspects of investigation, including disease modeling, target identification, and treatment development. While some reviews have effectively summarized recent technological advances in organoids, few have explicitly explained how these innovations can be applied to disease scenarios. Therefore, we hope this review will help researchers better utilize organoids by providing application frameworks for specific NDDs, deepening their understanding, and advancing insights into the pathophysiology of NDDs. The timeline is generally shown in **Figure 1**.

## Search Strategy

We searched the PubMed database using keywords such as “organoid,” “neurodegenerative diseases,” “Alzheimer’s disease,” “Parkinson’s disease,” “Huntington’s disease,” “amyotrophic lateral sclerosis,” “frontotemporal dementia,” “assembloid,” “microfluidic technologies,” “bioprinting,” and “transplantation.” We primarily focused on articles published in the last 5 years while also referencing milestone articles. In total, we reviewed 194 articles on AD, 132 on PD, 18 on HD, 31 on ALS, and 26 on FTD. Overall, 264 articles were included in this review. The details are shown in **Additional Table 1**.

**Additional Table 1:** Primary searching results

Disease	Total	Culture	Microfluidic technologies	Bioprinting	Transplantation	Assembloids
AD	194	61	81	9	10	6
PD	132	44	40	4	16	5
HD	18	4	4	1	1	2
ALS	31	11	20	1	1	0
FTD	26	7	1	0	2	0

AD: Alzheimer's disease; ALS: amyotrophic lateral sclerosis; FTD: frontotemporal dementia; HD: Huntington's disease; PD: Parkinson's disease.

## Construction and Limitations of Classic Brain Organoids

### Construction of brain organoids

In 1992, researchers at the University of Calgary Faculty of Medicine created an entirely new *in vitro* model to aid their investigation. After removing striata from CD-1 albino mouse embryos, they dispersed the tissue and inoculated it into serum-free medium with various added substances, such as insulin, transferrin, and epidermal growth factor. This resulted in an *in vitro* tissue model displaying features similar to the striatal region, which can be considered an early-stage organoid analog (Reynolds et al., 1992). However, this method did not immediately attract widespread attention. It was not until 20 years later that the concept of organoids, in its true sense, was proposed. Japanese scientists pioneered the creation of an artificial optic cup, which showed stratified neural retinal tissue through 3D culture from mouse embryonic stem cell aggregates. They were surprisingly optimistic about the self-patterning of these cells in suspension culture, predicting it would be valuable for future central nervous system (CNS) research (Eiraku et al., 2011). Investigators in the United Kingdom then took the lead, starting with the induction of embryoid bodies (EBs) to neuroectodermal tissue, followed by culturing in a 3D environment and encapsulating the tissue in Matrigel droplets. This provides a scaffold capable of supporting the development of more complex tissues (Lancaster et al., 2013). The applications of this approach were later expanded by additional studies. For example, single-cell RNA sequencing revealed impaired production of excitatory neurons in Williams syndrome forebrain organoids (Zhao et al., 2024). Bulk and single-cell transcriptomic profiling of fragile X syndrome forebrain organoids showed that the loss of fragile X mental retardation protein causes cell type-specific transcriptional dysregulation (Kang et al., 2021).

Fundamentally, the classical organoid culture method is based on dissociating induced pluripotent stem cells (iPSCs) into individual cells using mechanical, enzymatic, or immunomagnetic bead-based techniques (Ji and Sun, 2024). Afterwards, these isolated cells are reaggregated into specific sizes and cultured within a supportive matrix such as Matrigel or tissue extracellular matrix hydrogels (Wu et al., 2024b; Lai et al., 2025). Adding various bioactive substances to the 3D culture medium helps the organoids mature. Researchers then collect these organoids for further analysis and study (Gu et al., 2023). However, despite this traditional approach, several challenges still need to be addressed.

### Limitations of current organoids

#### Lack of certain cell types

Classic organoids lack several specific cell types, particularly neuroglial cells (Urrestizala-Arenaza et al., 2024). For example, microglia play a crucial role in supporting functional connectivity during brain differentiation (Pramanik et al., 2024). They are responsible for immune surveillance in the brain under normal physiological conditions during the resting state (Hu et al., 2025). This duration is significant in NDDs. In AD, microglia are thought to cause excessive damage to neuronal synapses, which can lead to neurodegenerative outcomes (De Schepper et al., 2023). In PD, microglia can contribute to the death of many neurons by promoting inflammatory responses (Chakraborty et al., 2023). Therefore, reproducing microglia in organoids is necessary to improve reproducibility (Kalpana et al., 2025).

#### Lack of blood–brain barrier structure

At the tissue level, conventional organoids lack perfusable microvascular networks, which is one of the main reasons why their representativeness remains questionable in the eyes of many scholars, as the relationship with neurons is too close to the vascular system (Tan et al., 2023). The terms “neurovascular unit” (NVU) or “neurovascular complex” have been introduced to emphasize this close connection (Zhou et al., 2023). Hemodynamic flow supplies oxygen and nutrients to neural stem cells (NSCs) while removing metabolic waste, thereby supporting neural development and differentiation (Genet et al., 2023; Pfau et al., 2024). In fact, purely original organoids without vasculature are significantly smaller, highly cytotoxic, and tend to develop a core of necrotic cells when cultured for an extended period (Lancaster et al., 2013).

#### Low reproducibility

Standardization is one of the challenges associated with diverse protocols and the use of different materials (El Harane et al., 2025). Matrigel, which is derived from mouse sarcoma, is a mixture of various extracellular matrix (ECM) components that exhibit inconsistent reproducibility. As a heterogeneous cocktail of factors, its standardization is exceptionally difficult (Carpentier et al., 2024). A variety of alternatives are currently in development. For example, a cationic peptide amphiphile containing laminin-based amino acid sequences has adjustable stiffness, which can be modified through co-assembly with negatively charged tyramine-modified hyaluronic acid. After 4 days of culture, the EBs grown on this matrix showed increased growth and maturity. It is also important to note that, since this material lacks any biological origin, it offers more stable standardization compared with other biological materials (Isik et al., 2023).

In conclusion, many challenges still need to be addressed innovatively, such as the lack of adult maturation and aging processes, the absence of a blood–brain barrier (BBB), and the complexities of organoid engineering (Jeong et al., 2023).

## Advances in Organoid Technology

**Figure 2:**
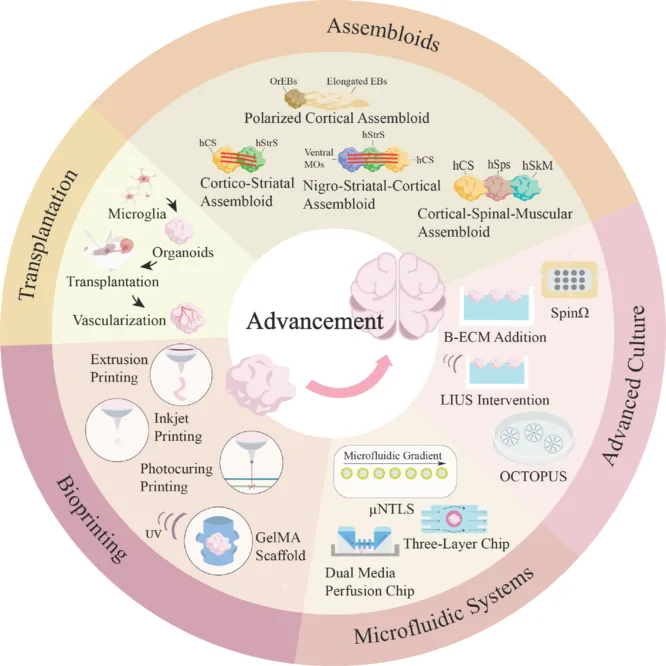
Advancements of organoids. B-ECM: Brain extracellular matrix; EBs: embryoid bodies; GelMA: gelatin methacrylate; hCS: human cortical spheroids; hSkM: human skeletal muscle spheroids; hSpS: human spinal spheroids; hStrS: human striatal spheroids; LIUS: low-intensity ultrasound; MOs: midbrain organoids; OrEBs: organizer-like embryoid bodies; UV: ultraviolet; µNTLS: microfluidic neural tube-like structure.

Beyond the previously mentioned concerns, researchers in this field have developed a diverse range of sophisticated approaches to address these issues and advance the development of organoids. We conducted a thorough review of the different methodologies used to enhance organoid development, primarily focusing on advanced culture techniques, microfluidic systems, bioprinting, transplantation, and assembloids (**Figure 2**).

### Advanced culture

As previously reviewed, the general culture of organoids is primarily conducted using the Matrigel-based method, also known as the “dome culture method” (Co et al., 2023). However, there are ongoing limitations, including the limited robustness of experiments, complex operational routines, and insufficient control throughout the culture procedure. To effectively address these limitations, several high-performance culture techniques have been developed, such as suspension culture (Yang et al., 2024) and air-liquid interface culture (Cañizares Luna et al., 2025).

#### Innovation of cultural devices

The innovations in cultural devices are highly significant. Bioreactors have been prominently developed, such as SpinΩ. This device is designed based on the 12-well tissue culture plate and utilizes 3D printing to create specialized organoid suspension chambers. This approach provides a more tailored cultivation platform for producing organoids compared with traditional culture flasks, which often result in significant waste of cultivation factors. Furthermore, the organoids grown in this system exhibited more mature layers and a clearly defined outer subventricular zone compared with those generated in spin flasks (Qian et al., 2018). This device was also used to generate Zika virus-infected organoids, demonstrating its utility as a model system in neurological research for disease modeling and target identification (Qian et al., 2016).

Another uniquely innovative device, known as “OCTOPUS,” facilitates the culture of organoids within an open microfluidic system. This microfluidic system features a radially symmetric architecture comprising eight modular culture compartments. Each chamber radiates from a shared central reservoir, which includes an unobstructed inlet/outlet port, thereby establishing continuous perfusion dynamics that promote the maturation of organoids (Park et al., 2022).

#### Engineered matrix

The culture medium, as the immediate source of nutrition for organoid maintenance, is another key determinant in organoid culture systems. A decellularized brain ECM hydrogel (B-ECM hydrogel) has been proposed as an appealing choice. Previous studies have used an innovative detergent and enzyme-based approach to decellularize porcine tissues, resulting in the production of a B-ECM hydrogel (Simsa et al., 2021; Kellaway et al., 2023). By recapitulating the microenvironment of the human brain, engineered ECMs enhance the fidelity of organoid-based modeling for genuine pathological states. Human brain ECM (B-ECM) co-culture, when incorporated into a Matrigel-based organoid culture, significantly increased cellular proliferation and facilitated more desirable intercellular interactions during the early phases of neurogenesis (Cho et al., 2021). This decellularization process demonstrates that the hydrogel preserves organoid culture properties similar to Matrigel, while the residual DNA content is negligible (Simsa et al., 2021).

#### Physical cues

Understanding physical microenvironmental signals is critical for uncovering the mechanisms driving neurogenesis and neural development. However, existing organoid technologies have largely failed to reconstruct a neuronal physical microenvironment that faithfully replicates the biomechanical and structural features of native neural tissues (Li et al., 2024a). In a prior study, Li et al. (2024b) used low-intensity ultrasound (LIUS), a technique previously applied in two-dimensional models to enhance cell differentiation and proliferation, treating it as a mechanical stimulus. They investigated whether LIUS could induce the same effects in 3D culture conditions. Yes-associated protein (YAP) is a mechanosensitive transcriptional activator that is activated upon nuclear import and regulates downstream effector transcription, driving mitotic progression (Driskill and Pan, 2023). Western blot analysis showed that YAP was more localized in the nucleus and less expressed in the cytoplasm in LIUS-induced organoids. Knockdown of YAP reversed the LIUS-stimulated proliferation, indicating that YAP plays an indispensable role in the proliferation of organoids treated with LIUS (Li et al., 2024b).

In conclusion, benefiting from diverse innovations, including well-designed devices (Qian et al., 2016), engineered ECMs (Cho et al., 2021), and physical modulation approaches (Li et al., 2024b), advanced culturing methodologies partly addressed the problems that hinder classical culture protocols.

### Microfluidic systems

Although great advances have been made in developing organoids that are physiologically representative, they still share common shortcomings due to being situated in a relatively static matrix and lacking key components of organ function found *in vivo* (Liu et al., 2024a; Li et al., 2025). Importantly, the absence of dynamic interstitial perfusion and spatiotemporally programmed biomechanical stress gradients, which are characteristic of physiologically intact *in vivo* niches, presents a core barrier to successful translational modeling of organs (Yu et al., 2025). Microfluidic systems have been developed to address this challenge, giving rise to the concept of “organoids on chips” (Bhatia and Ingber, 2014; Liu et al., 2024a). A fundamental microfluidic chip can be envisioned as a microscale unit designed for cell culture, characterized by a channel formed using etched silicon wafers as molds (Bhatia and Ingber, 2014).

#### Precision-programmed microfluidic systems

Microfluidic systems can precisely control the growth environment of organoids, thereby allowing for the construction of more complex and refined organoid models (Abdulla et al., 2023). The neural tube, which begins as a tubular primordium during embryogenesis, serves as the embryonic progenitor of the CNS, encompassing the primordia of the brain and spinal cord (Zeng et al., 2023). Investigators have developed a microfluidic neural tube-like structure to replicate the morphogenesis and regionalization of the embryonic neural tube (Xue et al., 2024; Luo et al., 2025). By combining microcontact printing, they fabricated arrays of Geltrex adhesive islands containing basement membrane proteins found in the microenvironment of the neural tube. This led to the assembly of human pluripotent stem cells (hPSCs) into defined tubular structures approximating neural tube geometry. Simultaneously, they developed a microfluidic setup that allowed them to create chemical gradients at will. To provide the right-side reservoir of the central channel with the WNT pathway activator CHIR99021, fibroblast growth factor 8 (FGF8), and retinoic acid (RA), a gradient along the rostral-caudal axis was established. Additionally, a gradient along the dorsal-ventral axis was achieved by filling the top and bottom channels with bone morphogenetic protein 4 and sonic hedgehog (SHH) signaling molecules. Their strategy successfully reproduced the intricate spatial pattern of neural tube morphogenesis (Xue et al., 2024).

#### Diverse structures

The architectural designs of these devices are highly diverse, depending on the targeted environment. One example of such a system comprises three layers, with each layer containing five fluidically coupled chambers connected by microchannels to maintain fluid perfusion and facilitate dynamic exchange of media between compartments. The simplicity of the fluidic compatibility between the apparatus and peristaltic pumps or microfluidic manifolds allows the system to be highly parallelizable and fully multiplexed using a common orbital agitation platform, thereby facilitating multiplexed phenotypic profiling (Cho et al., 2021). Another device features a circular lattice microarchitecture with two media perfusion channels that guide organoid morphogenesis. This design enables biomimetic flow-mediated cell transport of dissolved gases and metabolites, as well as differential flow shear stresses to trigger leukocyte chemotaxis around the edges of the organoid (Ao et al., 2022).

In conclusion, microfluidic devices support organoid construction in a more controllable manner, and their structure can be specifically adjusted to meet unique requirements.

### Bioprinting

Bioprinting is a novel biofabrication process that creates biologically active tissues through precisely controllable methods for the spatial patterning of cells and biological materials (Maharjan et al., 2024). It can fabricate models of any shape with strict reproducibility based on a specific computer program (Juraski et al., 2023). The general bioprinting techniques used include inkjet printing, extrusion printing, and photocuring printing (Frankowski et al., 2023). Given the diverse organizations of organoids, bioprinting techniques are expected to offer various tailor-made opportunities for organoid generation (Maharjan et al., 2024). A variety of materials are utilized to provide organoids with adjustable supportive scaffolds exhibiting tunable physical properties, thereby enhancing the maturation trajectory and structural-functional fidelity of the bioengineered constructs (Mancuso et al., 2024). Researchers can customize the culture matrix by incorporating other substances into Matrigel or simply replacing it. Gelatin Methacrylate (GelMA) is synthesized through the chemical modification of gelatin macromonomers via methacryloyl substitution (Kjar et al., 2024). This photo-crosslinkable biomaterial exhibits exceptional cytocompatibility, thereby offering a biomimetic ECM niche that facilitates cellular anchorage, mitotic expansion, and lineage specification. By adjusting its concentration, temperature, and cross-linking conditions using ultraviolet light, the mechanical properties of GelMA can be effectively tailored. Within additive biofabrication frameworks, GelMA serves as a principal bioink matrix. The programmatic regulation of viscoelastic parameters enables micrometer-resolution patterning of cellularized hydrogel constructs while permitting layer-by-layer assembly of hierarchical 3D tissue architectures (Wang et al., 2024).

### Transplantation of organoids

According to the literature, inadequate oxygen and nutrient supply are intrinsic faults of classic *in vitro* culture systems, leading to prolonged cellular stress responses and necrotic core formation within organoid architecture (Xu et al., 2024b). To address this issue, Wang et al. (2023) developed a transplantation technology that involves transplanting brain organoids into animal subjects and introducing specialized cell types into the organoids. This technique allows organoids to achieve adequate vascularization through the surrounding host tissue.

#### Transplantation at the tissue level

A trial involving the transplantation of organoids into the primary somatosensory cortex (S1) of early postnatal athymic rats demonstrated remarkable success. The transplantation protocol was applied during postnatal days 3 to 7, a developmental stage when thalamocortical and corticocortical axonal projections have not yet fully innervated S1, thereby facilitating improved integration of the organoids. The average size of the transplanted organoids increased ninefold. Compared with those cultured *in vitro*, the transplanted organoids exhibited higher expression levels of brain-derived neurotrophic factor, secretogranin II, and osteocrin, along with upregulation of transcripts associated with synaptogenesis, astrogenesis, and myelination (Revah et al., 2022). Specifically, astrocytic projections form specialized contact points with cerebral microvasculature through polarized perivascular terminals known as endfeet. The molecular machinery comprising aquaporin 4 water channels and Kir4.1 potassium transporters becomes compartmentalized within these astrocytic endfoot domains, orchestrating the regulation of fluid-electrolyte gradients across the NVU and enhancing cell maturation in engrafted organoids. Furthermore, engraftment in immunodeficient rats triggered elevated expression levels of gliogenic activation markers such as glial fibrillary acidic protein and aquaporin 4, resulting in enhanced cellular diversity (Wang et al., 2025). The transplantation site significantly impacts the characteristics of the organoids. Some researchers have grafted organoids into the prefrontal cortex and hippocampus. Organoids in the hippocampus favor glial and neuronal maturation, while organoids in the prefrontal cortex demonstrate stronger astrocytic function and earlier maturity, which is attributed to the higher density of dopaminergic and cholinergic neurons in the prefrontal cortex (Xu et al., 2024c).

#### Transplantation at the cellular level

Besides tissue-level transplantation, cell-level transplantation is also a critical aspect. Microglia are derived from yolk sac-derived erythromyeloid progenitors (Schafer et al., 2023). As mentioned in the section “Limitations of current organoids,” generating microglia in organoids poses a significant challenge. To address the deficiency of microglia in conventional organoids, a specific type of organoid was co-cultured with erythromyeloid progenitors. These organoids were subsequently implanted into the retrosplenial cortex after 10 days of co-culture. Successful vascularization occurred, providing adequate support for the tissue. The transcriptional signature exhibited elevated expression levels of neuronal specification markers SATB homeobox 2 (SATB2) and T-Box brain transcription factor 1 (TBR1), along with astrocytic functional genes S100B and solute carrier family one member 3 (EAAT1), reflecting coordinated neuroglial maturation dynamics. Longitudinal tracking of graft surface area during the first 2 weeks revealed an initial decrease in tissue size before vascularization, which was followed by subsequent expansion that temporally coincided with the formation of the vascular system (Schafer et al., 2023).

Transplanting tissues or individual cells advances organoid research in live models by addressing integration at different scales: system-wide connectivity at the macro level versus fine-tuned control of cellular organization at the micro level. Tissue transplantation helps engrafted material functionally adapt by recreating authentic neural development environment. Meanwhile, cell-level transplantation reveals how specific cell lineages communicate by precisely adjusting signaling between cells (Dill-Macky et al., 2024; Xu et al., 2024c; Pașca et al., 2025).

### Assembloid models

The classical organoids are specifically developed to mimic a certain region of the brain, tryings to reproduce the exact molecular phenotype and physiological function. However, due to the complex communication of regions in the brain and diverse cell types leading to microenvironment with spatial characteristics, natural organs present a higher level of information storage capacity than classic organoids (Kim and Chang, 2023). Although derived from iPSCs, assembloids containing further information are composite models constructed by integrating distinct organoids with different inducing patterns, often in conjunction with specialized cells. This method can replicate the natural communication between brain regions, hence allowing the creation of a more authentic 3D architecture (Onesto et al., 2024).

#### Polarized cortical assembloids

Glutamatergic projection neurons and GABAergic interneurons represent the predominant neuronal populations in the human brain, originating from dorsal and ventral forebrain progenitors, respectively (Ren et al., 2024a; Zhai et al., 2024). In a recent study, researchers engineered polarized cortical assembloids by integrating FGF8-expressing organizer-like embryoid bodies with geometrically constrained elongating hPSC-derived organoids. The protocol entailed culturing hPSCs within 15-mm polydimethylsiloxane (PDMS) micro-molds to generate axially patterned organoids that were subsequently immobilized in Matrigel to preserve morphological polarity. This spatially confined system facilitated sustained establishment of an FGF8 gradient through paracrine secretion from organizer-like embryoid bodies, effectively recapitulating molecular hallmarks of rostrocaudal axis patterning during human corticogenesis (Bosone et al., 2024).

#### Cortico-striatal assembloids

Cortico-striatal projections mediate human motivation. To elucidate this pathway and probe its dysfunction, researchers constructed assembloids of human cortical spheroids (hCS) and human striatal spheroids (hStrS) *in vivo*, using the enhanced yellow fluorescent protein marker as an indicator for the cerebral cortical organoid. This was achieved by establishing a unilateral connection between hCS and hStrS. hCS was thereafter infected with hSyn1::ChrimsonR-tdTomato for optogenetic stimulation with the subsequent calcium imaging analysis. It demonstrates that hStrS cells showed calcium responses to tdTomato after hCS was irradiated by a red-shifted light of 625 nm, which confirmed the formation of a functional connection and identified the participation of medium spiny neurons (Miura et al., 2020).

#### Nigro-striatal-cortical assembloids

The substantia nigra is a very important part of the midbrain, associated with various NDDs (Zheng et al., 2023; Chu et al., 2024; Lin et al., 2025; Tang et al., 2025). Nigro-striatal-cortical assembloids emerge as a notable model for investigation. The assembloid was made up of three distinct organoids in a PDMS mold with a certain spatial arrangement order, as the ventral midbrain organoid and the cortical organoid set in the digitals, with the striatal organoid in the middle. Through fusion, the distinct organoids became an entirety with functional neural networks validated by optogenetic stimulation, with mature cells detected by single-cell RNA sequencing (Reumann et al., 2023).

#### Cortical-spinal-muscular assembloids

The cortico-motor pathway plays an important role in physical function, while its despair matters a lot in some kinds of NDDs such as ALS (Yang et al., 2025). Step by step, cortical-spinal-muscular assembloids were created to be a research platform. Using a modular assemblage paradigm, iPSCs were sequentially patterned to generate hCS, human spinal spheroids (hSpS), and human skeletal muscle spheroids (hSkM). COs were transduced with an adeno-associated virus (AAV) encoding the optogenetic actuator ChrimsonR-tdTomato under the synapsin promoter, while hSkM were engineered for real-time myofiber activity monitoring. The three components were spatially oriented on a permeable Transwell insert in a stepwise manner—hSpS and hSkM were first juxtaposed to establish neuromuscular connectivity, followed by precise apposition of COs. This construct supports multilevel synaptic transmission from cortical neurons to spinal motor neurons and subsequently to skeletal muscle fibers, enabling optogenetically evoked and quantifiable muscle contractions (Andersen et al., 2020).

## Application of Organoids in Neurodegenerative Diseases

NDDs are a group of disorders characterized by the progressive degeneration of neurons, leading to cognitive decline, motor dysfunction, and loss of autonomy. This group includes AD, PD, HD, ALS, and FTD (Wilson et al., 2023). Organoid technology was swiftly adopted for NDD research following its conceptual emergence (Lei et al., 2024). Researchers have successfully integrated multiple technologies to enhance the functional completeness of organoid models. In the following sections, we will introduce the specific applications of organoids in certain diseases, including disease modeling, target discovery, and therapeutic strategies.

### Alzheimer’s disease

AD is a progressive neurodegenerative dementia characterized pathologically by the presence of extracellular amyloid-β (Aβ) plaques and intracellular neurofibrillary tau tangles (Twarowski and Herbet, 2023; Long et al., 2025; Luo et al., 2025). These molecular lesions contribute to the progressive decline in neurocognitive function, which includes episodic memory impairment, executive dysfunction, and various neuropsychiatric manifestations (Maji and Khajanchi, 2025). Organoid models have garnered substantial research attention due to their unique capacity to recapitulate patient-specific pathophysiological hallmarks. These models have attracted significant interest for their ability to replicate patient-specific features (Karmirian et al., 2023; Scopa et al., 2023).

#### Disease modeling

Key pathological processes in AD include Aβ overexpression and fibrillar deposition, along with neurofibrillary tangles composed of hyperphosphorylated tau. COs have emerged as widely adopted models for AD, effectively reproducing these pathological features (Liu et al., 2024b; Zhang et al., 2024). Researchers first transfected ReNcell VM human NSCs (ReN cells) and engineered a polycistronic lentivirus to introduce the amyloid precursor protein (*APP*) and presenilin 1 (*PSEN1*) genes. They used BD Matrigel, which is rich in brain ECM proteins, as a supporting matrix. As the organoids matured, the expression levels of adult 4-repeat tau isoforms peaked, indicating enhanced fidelity in representing AD pathology. These cells also exhibited higher concentrations of total tau and hyperphosphorylated tau within the same cells, displaying abnormal morphologies with beaded processes similar to those found in the dystrophic neurites of AD brains (Choi et al., 2014).

**Figure 3:**
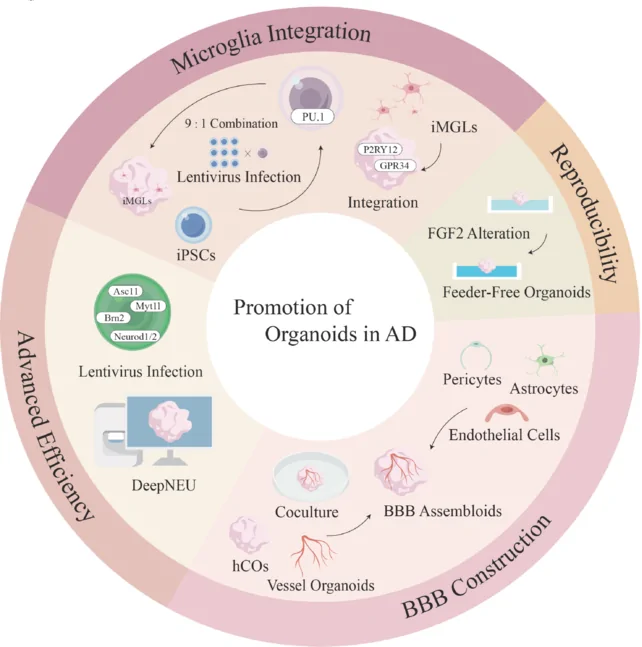
Promotion of organoid construction in Alzheimer’s disease (AD). AD: Alzheimer’s disease; Ascl1: achaete-scute homolog 1; BBB: blood–brain barrier; Brn2: POU domain transcription factor; FGF2: fibroblast growth factor 2; GRP34: G protein-coupled receptor 34; hCOs: human cerebral organoids; iMGLs: iPSC-derived microglia-like cells; iPSCs: induced pluripotent stem cells; Myt1l: myelin; P2Y12: purinergic receptor P2Y12.

In the advancement of COs (**Figure 3** and **Additional Table 2**), researchers have focused on enhancing the reproducibility of CO generation through standardized protocols. Minimizing uncontrollable biological factors in the production process is critical. The biological origin of feeder layer cells can lead to reproducibility issues. In contrast, some researchers have successfully developed feeder-free organoids by modulating the concentration of FGF2 in the culture media (Shimada et al., 2022).

**Additional Table 2:** Promotion of organoids in AD

Disease	Promotion	Approach	Reference
Alzheimer's disease	Advanced efficiency	Fibroblasts expressing Ascl1, Brn2, Myt1l, and neurod1/2 were directly cultured within Matrigel after transfecting them with specifically designed lentiviral particles.	Kim et al., 2024b
		DeepNEU is a framework driven by deep learning, specifically designed to simulate the dynamics of iPSCs through multilayer perceptron architectures.	Esmail and Danter, 2021
	Reproducibility	Feeder-free organoids are constructed by modulating FGF2 concentration in the culture media, eliminating the need for a feeder layer system.	Shimada et al., 2022
	BBB construction	IPSCs were initially differentiated into endothelial cells, pericytes, and astrocytes. Subsequently, these cell types were combined in Matrigel supplemented with 10% fetal bovine serum and growth factors.	Blanchard et al., 2020
		Initially, vascular organoids were cultured within a matrix composed of collagen type I and Matrigel. Subsequently, these organoids were co-cultured with cortical organoids undergoing differentiation to promote fusion.	Kong et al., 2023
	Microglia intergration	HPCs were typically derived from iPSCs using the STEMdiff Hematopoietic Kit and subsequently differentiated into iMGLs in a specialized medium.	Park et al., 2024
		Lentiviral vectors were used to induce PU.1 expression in hESCs. Subsequently, 10% of PU.l-infected hESCs were mixed with 90% parental hESCs to generate COs.	Cakir et al., 2022

AD: Alzheimer's disease; Ascl1: achaete-scute homolog 1; BBB: blood-brain barrier; Brn2: POU domain transcription factor; COs: cerebral organoids; FGF2: fibroblast growth factor 2; hESCs: human embryonic stem cells; HPCs: hematopoietic progenitor cells; iMGLs: iPSC-derived microglia-like cells; iPSCs: induced pluripotent stem cells; Myt1l: myelin.

In addition to improving the reproducibility of organoids, improving the efficiency is also very important. Waiting for neurons to mature can be frustrating. Precision control over organoid maturation phases enables a significant reduction in experimental timelines, accelerating discovery without compromising model validity (Kim et al., 2024b). Kim et al. (2024b) developed a method that circumvents the maturation period typically required for fibroblast differentiation. They directly cultured fibroblasts expressing achaete-scute homolog 1 (Ascl1), POU domain transcription factor (Brn2), myelin transcription factor 1-like (Myt1l), and neurod1/2 within Matrigel after transfecting them with specifically designed lentiviral particles. This approach was subsequently used in investigations related to apolipoprotein Z (APOE). When real-life tissues require a long-term culturing process, directly simulating with computer technology is a good alternative. DeepNEU represents a computational framework driven by deep learning, specifically designed to simulate the dynamics of iPSCs through multilayer perceptron architectures. This framework integrates epigenetic remodeling algorithms with Boolean network modeling to effectively recapitulate the trajectories of pluripotency acquisition and lineage commitment. Esmail and Danter (2021) used this approach to construct a virtual AD organoid, which accurately predicts the characteristics of various brain regions as well as AD-related pathologies.

As mentioned in the chapter “Limitations of current organoids,” organoids lack the vascular infrastructure necessary for studying the contribution of vascular factors, a known hazard in AD (Wang et al., 2021; Xie et al., 2025). This methodological constraint has been addressed through the development of assembloid technology (Takeuchi et al., 2025). In 2020, researchers produced BBB organoids with an upregulated molecular phenotype by first differentiating iPSCs into endothelial cells, pericytes, and astrocytes, before incorporating these three cell types into Matrigel with 10% fetal bovine serum (FBS) and growth factors (Blanchard et al., 2020). Additionally, brain vasculature organoids have been used to explore the involvement of SARS-CoV-2 in AD. These organoids are initially grown in a collagen type I matrix with Matrigel and then co-cultured with cortical organoids to facilitate fusion (Kong et al., 2023).

As previously mentioned in “Limitations of current organoids,” non-neuronal cells play a crucial role in the pathologies associated with AD. Notably, astrocyte-like cells naturally emerge during the culture of COs (Quadrato et al., 2017). Consequently, researchers do not need to focus on generating these cells but should instead concentrate on promoting their further maturation (Wang et al., 2025). In contrast, the absence of microglia poses a more significant challenge due to their distinct origin compared with neurons and astrocytes (Sun et al., 2023). Microglial proliferation and activation, especially around amyloid plaques, are hallmarks of AD, making it necessary to incorporate microglia into COs (Park et al., 2024). Various methodologies have been used to address this issue. Human iPSC-derived microglia-like cells (iMGLs) have been successfully integrated into brain organoids, where they exhibit characteristics similar to *in vivo* microglia, including the expression of canonical microglial markers such as purinergic receptor P2Y12 (P2RY12) and G protein-coupled receptor 34 (GPR34), serving as an alternative to natural cells (Abud et al., 2017). Hematopoietic progenitor cells are typically derived from iPSCs using the STEMdiff Hematopoietic Kit and subsequently differentiated into iMGLs in a specialized medium (Park et al., 2024). Recently, the transcription factor PU.1 has emerged as a viable option for generating microglia within organoids. Lentiviral vectors were used to induce PU.1 expression in human embryonic stem cells (hESCs). Subsequently, 10% of PU.1-infected BC4 hESCs were mixed with 90% parental hESCs to generate COs. The experimental organoids demonstrated a higher spontaneous firing frequency compared with the control group, highlighting the role of microglia in promoting CNS maturation (Cakir et al., 2022).

#### Target discovery and therapeutic strategies of Alzheimer’s disease

Because of these 3D *in vitro* structures that imitate essential features of *in vivo* tissue environments, organoids constitute a valuable translational link between reductionist 2D monolayer models and animal models (Zeldich and Rajkumar, 2024). ATP Binding Cassette Subfamily A Member 7 (ABCA7) is one of the significant biologically related process genes. Mitochondrial enlargement and oxidative stress appear in organoids derived from iPSC ABCA7-deficient cells, and these dysfunctions are rescued by the supplementation of phosphatidylglycerol, suggesting the essential role of ABCA7 in regulating mitochondrial lipid metabolism to prevent Alzheimer’s disease pathology (Kawatani et al., 2024).

Organoids demonstrate that many non-genetic factors contribute to AD pathologies (Lu et al., 2024). For instance, incubating cortical organoids with human serum for 12 days was shown to increase Aβ content, recapitulating features of AD. During this exposure period, beta-secretase 1 was upregulated compared to the control, resulting in increased cleavage of APP and, consequently, the generation of Aβ (Chen et al., 2021). Per- and polyfluoroalkyl substances (PFASs) are toxic organic chemicals commonly used in a wide range of applications. Studies involving brain organoids exposed to PFAS compounds such as perfluorooctanoic acid (PFOA), perfluorooctane sulfonate (PFOS), and perfluorohexane-sulphonic acid (PFHxS) have revealed increased accumulation of Aβ and hyperphosphorylation of tau proteins (Delcourt et al., 2024; Lu et al., 2024).

Most infectious agents can exacerbate the disease process of AD (Xie et al., 2023; Beydoun et al., 2024). Aβ and tau phosphorylation were found in COs infected with HSV-1 (Hyde et al., 2025). Notably, there is a discrepancy in the distribution of Aβ between 2D models and COs. In 2D models, Aβ aggregates inside HSV-1-infected cells, while in COs, Aβ aggregates are located in cells surrounding the HSV-1-infected cells. Given that 3D models can provide more comprehensive data, the results obtained from COs should be considered more reliable than those from 2D models (Abrahamson et al., 2021). Further mechanisms were elucidated in human brain organoids, where HSV-1 infection increased tau phosphorylation; this phosphorylation reduced neuronal death after infection, suggesting that tau phosphorylation might serve as an immune defense mechanism against HSV-1 infection (Hyde et al., 2025).

Given the manipulability of organoids and their high level of personalization derived from patient-specific sources, organoids are widely utilized in therapeutic development, offering significant advancements in refining treatment strategies for AD (Nemergut et al., 2023; Zhang et al., 2024). Semaglutide, a hormone-based medication used for managing type 2 diabetes as a glucagon-like peptide-1 (GLP-1) receptor agonist, lowers blood glucose levels by mimicking the action of insulin in the human body (Karagiannis et al., 2024). In a recent investigation, researchers assessed the potential efficacy of semaglutide in AD by exposing COs derived from AD patients to human serum, thereby simulating the pathological environment characteristic of the disease. Following a 5-day treatment with semaglutide, these organoids demonstrated significantly elevated expression levels of oxytocin (OXT), attributed to the activation of the GLP-1 receptor. This increase in OXT expression subsequently resulted in a notable reduction in glial fibrillary acidic protein levels within astrocytes, suggesting a possible neuroprotective effect (Zhang et al., 2024). Tramiprosate and its metabolite 3-sulfopropanoic acid induce an ApoE3-like conformation in ApoE4 isoforms, thereby diminishing their aggregation propensity. In ApoE4-derived COs, tramiprosate treatment modulates cholesteryl ester levels, a marker indicative of cholesterol storage, suggesting enhanced lipid metabolism (Nemergut et al., 2023).

### Parkinson’s disease

PD is characterized as a multisystem proteinopathy, primarily defined by the selective degeneration of mesencephalic dopaminergic circuitry, particularly affecting dopaminergic neurons within the substantia nigra pars compacta (Tanner and Ostrem, 2024). This neurodegeneration is pathologically marked by the accumulation of LBs, eosinophilic cytoplasmic inclusions composed of misfolded α-syn fibrillary assemblies, alongside progressive neuromelanin depletion (Tong et al., 2024). The α-syn induced neurodegenerative cascade results in early dopaminergic axonal degeneration, occurring well in advance of somatic neuronal death, and ultimately presents as the cardinal motor triad comprising bradykinesia, rigidity, and resting tremor (Tanner and Ostrem, 2024). The complex etiology makes the pathophysiological process of PD very complicated, and Tanner and Ostrem (2024) conduct studies through various means.

#### Disease modeling

In 2014, dopaminergic neurons were recognized as an early stage of 3D models with the potential for further studies of PD, inspiring subsequent research (Tieng et al., 2014). In 2016, EBs were treated with dual-SMAD inhibition factors, a WNT pathway activator, SHH, and FGF8 to create a midbrain-like pattern. Electrophysiological assessments confirmed the functionality of the mature dopaminergic neurons within these models (Jo et al., 2016). Midbrain organoids (MOs) derived from patients with SNCA gene triplication mutations also demonstrated success, resulting in excessive expression of α-synuclein (Mohamed et al., 2021). By introducing glucosylceramidase beta 1 (GBA1) knockout and reproducing SNCA triplication in cells, researchers generated GBA1^−/−^::SNCA O/E embryonic stem cell lines, which were subsequently utilized in the construction of MOs. Under the combined influence of these two risk factors, the organoids exhibited more characteristic pathological phenotypes, particularly the emergence of Lewy body-like inclusions (LBLIs). Although these inclusions were not as large as those found in natural conditions, they still represented a successful reproduction of LBs (Jo et al., 2021).

In summary, midbrain organoids faithfully recapitulate the cardinal pathological hallmarks of PD, including α-synuclein aggregation and dopaminergic neuron degeneration. This human-derived model system has been systematically validated and integrated into preclinical research pipelines as a pathomimetic platform for investigating PD pathogenesis and therapeutic interventions (Jo et al., 2021; Cui et al., 2024).

**Figure 4:**
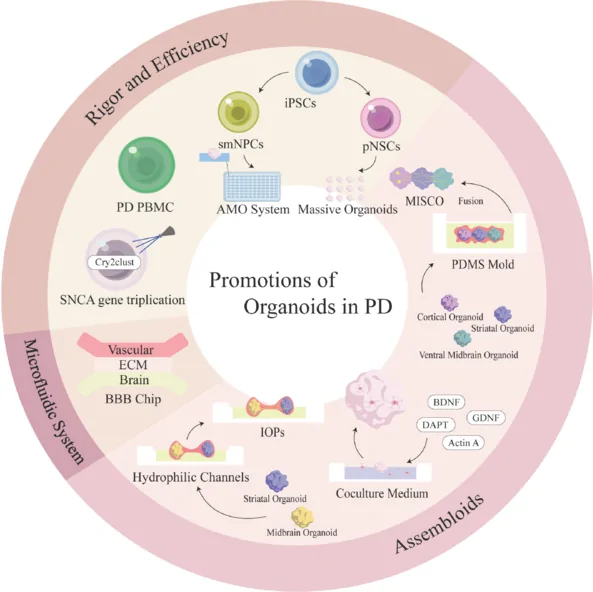
Promotion of organoid construction in Parkinson’s disease (PD). AMO: Automated midbrain organoid; BBB: blood–brain barrier; BDNF: brain-derived neurotrophic factor; DAPT: GSI-IX; ECM: extracellular matrix; GBA1: glucosylceramidase Beta 1; GDNF: glial cell line-derived neurotrophic factor; iPSCs: induced pluripotent stem cells; IOPs: inter organoid projections; MISCO: midbrain-striatum-cortex assembloid; PDMS: polydimethylsiloxane; pNSCs: proliferative neural stem cells; SNCA: synuclein alpha; smNPCs: small-molecule neural precursor cells.

While using organoids for PD research, scientists keep updating this versatile tool, and their achievements are shown in **Figure 4** and **Additional Table 3**.

**Additional Table 3:** Promotion of organoids in Parkinson's disease

Disease	Promotion	Approaches	Reference
Parkinson's Disease	Rigor and efficiency	SNCA-triplication iPSC-derived organoids replicate key PD features: Lewy body-like α-syn pathology, organelle injury, and fibril seeding.	Frattini et al., 2025
		By differentiating pNSCs in advance and storing them, researchers can directly initiate organoid formation from pNSCs in subsequent experiments.	Ha et al., 2020
		AMOs were developed by culturing smNPCs in a 96-well plate using an ECM-free protocol, reducing variability, improving reproducibility, and enabling high-throughput screening.	Renner et al., 2021
		Through putting GBA1 knock out and reproducing SNCA triplication together in cells, researchers harvest GBA1^-/-^::SNCA O/E ESC lines, which were subsequently utilized in MOs construction.	Jo et al., 2021
		The OASIS was developed using optogenetic technology. Researchers integrated Cry2clust, a blue light-responsive domain, into α-syn to induce protein homologous aggregation upon blue-light exposure. By applying blue light to iPSCs with triple SNCA expression, they created an organoid model for rapid α-syn aggregation, achieving significant temporal optimization in experimental model generation.	Kim et al., 2023
	Assembloids	MISCOs were constructed in a PDMS mold, as the three distinct organoids positioned within it in contact with Matrigel.	Reumann et al., 2023
		MP and SP spherical aggregates were placed in the hydrophilic regions of the culture surface. Matrigel was then added to cover the aggregates and hydrophilic channels between them, forming two mature, interconnected yet independent regions.	Ozgun et al., 2024
	Microglia integration	The coculture medium for microglia and organoids was advanced by incorporating BDNF, GDNF, DAPT, and Activin A into the microglia differentiation medium.	Sabate-Soler et al., 2022
	Microfluidic system	Through the spatially compartmentalized co-culture of vascular endothelial cells, neurons, and astrocytes within micro-engineered chambers designed to mimic physiological conditions, researchers successfully established a NVU microfluidic system.	de Rus Jacquet et al., 2023

AMOs: Automated midbrain organoids; BDNF: brain-derived neurotrophic factor; DAPT: GSI-IX; ECM: extracellular matrix; ESC: embryonic stem cell; GBA1: glucosylceramidase Beta 1; GDNF: glial cell line-derived neurotrophic factor; iPSC: induced pluripotent stem cells; MISCOs: midbrain-striatum-cortex assembloids; MOs: midbrain organoids; MP: midbrain precursor; OASIS: optogenetics-assisted α-syn aggregation induction system; PD: Parkinson's disease; PDMS: polydimethylsiloxane; pNSCs: proliferative neural stem cells; NVU: neurovascular unit; SNCA: synuclein alpha; smNPCs: small-molecule neural precursor cells; SP: Striatal precursor.

Rigor and efficiency are two essential components that cannot be overlooked. The strategic optimization of progenitor cell sources for organoid derivation significantly enhances organoid production efficiency by improving lineage-specific differentiation fidelity and reducing off-target cellular heterogeneity (Xu et al., 2024a). Currently, iPSCs obtained from patients with *SNCA* gene triplication have been utilized to generate organoids that can partially imitate characteristics of PD, including Lewy body–like α-synuclein pathology with diverse morphologies similar to Lewy bodies (LBs) found in patient cells. Additionally, the significant organelle damage detected through electron microscopy imaging and the seeding ability of α-synuclein fibrils also indicate the successful reproduction of PD pathology in these models (Frattini et al., 2025). By employing this strategy, researchers can construct PD organoids without relying on gene editing or chemical intervention techniques (Becerra-Calixto et al., 2023). These protocols initiate dopaminergic patterning induction at the onset of cell differentiation. In contrast, some scientists have utilized previous differentiation approaches to produce specific cell types for bulk organoid generation. By isolating proliferative NSCs (pNSCs) and storing them, researchers can use pNSCs directly for midbrain organoid-forming experiments later, thereby bypassing the time-consuming processes required to differentiate iPSCs (Ha et al., 2020). The construction of automated midbrain organoids (AMOs) represents an innovative technology in this field. Following this development, researchers successfully generated small-molecule neural precursor cells that exhibit ectoderm-restricted developmental potential and can be cultured in an ECM-free condition in a 96-well format. Currently, AMOs demonstrate less biological stochasticity, better reproducibility, and are ideal for high-throughput screening purposes (Renner et al., 2021). Furthermore, small molecules stimulate the WNT and SHH signaling pathways to direct ventral midbrain development. This novel method enhances human midbrain development, facilitating the production of dopamine neurons while also improving the maturity of non-neuronal cells, resulting in a more physiological *in vivo* model (Frattini et al., 2025). By providing designed physical cues to these cells, researchers can rapidly generate MOs. Optogenetic proteins are those whose activity can be modulated by light and are central to optogenetic technology. By precisely manipulating cellular activities through light, scientists can specifically control neuronal activities (Duan et al., 2025). The Optogenetics-assisted α-synuclein aggregation induction system was developed using optogenetic technology. Researchers incorporated Cry2clust, a light-responsive domain that reacts to blue-light stimulation, into α-synuclein. Aiming to accelerate the generation of α-synuclein aggregates, they created an organoid system using iPSCs with triple expression of SNCA exposed to blue light. This approach achieved a significant temporal improvement in the generation of experimental model systems (Kim et al., 2023).

Microfluidic systems enable precise recapitulation of PD pathophysiology by establishing well-defined culture conditions that mimic critical *in vivo* microenvironments, such as the NVU (de Rus Jacquet et al., 2023). Through the spatially compartmentalized co-culture of vascular endothelial cells, neurons, and astrocytes within micro-engineered chambers designed to replicate physiological conditions, researchers successfully established an NVU microfluidic system. This platform demonstrated that pharmacological inhibition of MEK1/2 signaling mitigated the pro-inflammatory phenotype of mutant astrocytes and restored BBB integrity (de Rus Jacquet et al., 2023).

In addition to more precise regulation of individual organoid production, researchers have adopted the method of generating assembloids to improve the simulation of physiological states in organoids (Reumann et al., 2023; Ozgun et al., 2024). Currently, PD organoids predominantly consist of midbrain organoids due to their high dopaminergic neuron content. However, PD symptoms affect multiple brain regions, such as the striatum and cerebral cortex, which are seldom replicated in organoids. The construction of assembloids is a promising approach for researchers, as it allows for regional customization to model neuroanatomical domains of interest (Barmpa et al., 2024). Ventral midbrain-striatum-cortex assembloids have recently emerged as a significant advancement in neurodevelopmental research. Researchers fabricated a PDMS mold, positioning three distinct organoids within it in contact with Matrigel, and subsequently transferred the assembly to an orbital shaker. By day 109, innervation from the ventral midbrain into the other two regions was observed to increase sharply, leading to the establishment of an integrated and functional neural network (Reumann et al., 2023). In another PD investigation, midbrain precursor (MP) and striatal precursor (SP) pellets were purposefully placed on the hydrophilic part of the culture surface, and Matrigel was deliberately added to cover the two spherical pellets and the hydrophobic gaps between them. These two types of tissues matured in separate chambers and were subsequently connected through a hydrophilic channel in the middle. This approach was particularly conducive to the development of mesencephalic and striatal organoids within heterotypic co-culture, thus enabling the assembly of assembloids. It is noteworthy that dopaminergic neurons and striatal GABAergic neurons formed synapses with each other autonomously via the dopaminergic chain through microelectrode array (MEA) technology. This platform enables longitudinal observation of neurodevelopment and stimulation-evoked network function. While calcium imaging showed spatially coherent firing in reciprocally connected organoids during the development of interorganoid projections (IOPs), this observed excitatory response corroborates the formation of functional synapses (Ozgun et al., 2024).

Similar to all classical organoids, midbrain organoids (MOs) are devoid of microglia, a condition that results in suboptimal fidelity of natural information and makes their representativeness far from satisfactory, thus exerting a significant impact on related research and analysis. Therefore, incorporating microglia into MOs would enable more accurate modeling of PDpathological states (Babu et al., 2024). In 2022, researchers assessed the interactions between microglia and MOs within their respective culture media. This work further advanced the co-culture medium for microglia and organoids by incorporating brain-derived neurotrophic factor, glial cell line-derived neurotrophic factor, DAPT, and activin A into the microglia differentiation medium. Immunostaining with ionized calcium-binding adapter molecule 1 (IBA1) confirmed the presence of microglia, while single-nucleus RNA sequencing analysis demonstrated microglial migration. Notably, these microglia exhibited typical immune functions that promote synaptic plasticity and enhance cell survival (Sabate-Soler et al., 2022).

#### Target discovery and therapeutic strategies of Parkinson’s disease

Mitochondrial genomics is a hot topic in PD, where MOs can dramatically shift the paradigm of experimental exploration of the underlying mechanisms. Analyses of the structure and function of mitochondria have shown that mutations in Miro1 trigger the downregulation of tricarboxylic acid cycle and oxidative phosphorylation metabolic processes. These temporal events have been pathomechanistically demonstrated at the level of patient-derived MOs, which exhibit hallmark traits such as mitochondrial respiratory chain uncoupling and cristae remodeling (Zagare et al., 2025). Structural-function analyses demonstrate that the p.R272Q mutation in Miro1 within the EF-hand motif leads to a pathological uncoupling of calcium buffering capacity, which subsequently elicits calpain-mediated proteolytic processing of α-synuclein. This mechanistic cascade has been validated in patient-derived MOs, which exhibit hallmark abnormalities, including mitochondrial respiratory chain uncoupling and cristae remodeling (Chemla et al., 2025). The endogenous heterogeneity of astrocytes in MOs offers an autologous system to study astrocyte-driven neuroinflammatory pathways in PD progression. This endogenous glial genesis captures the chronology of astrocyte activation observed in human PD progression (Geng et al., 2023). Given the existence of astrocytes expressing Parkinsonism-associated deglycase (DJ1) mutations responsible for early-onset PD, DJ1 knockout midbrain organoids (DJ1-KO MOs) provide an ideal model system to investigate this gene (Morrone Parfitt et al., 2024). Recent studies have established that DJ1 functions as a deglycase, which is essential for maintaining homeostasis of advanced glycation end-products (Morrone Parfitt et al., 2024; Skou et al., 2024; Lee et al., 2025). Dysregulation of DJ1 can lead to protein glycation damage, particularly within astrocytes. The initiation of this cellular damage triggers trans-neuronal pathogenic processes and disrupts the glial bioenergetic coupling essential for neuronal circuit integrity (Morrone Parfitt et al., 2024).

In addition to the search for potential targets, organoids are increasingly being incorporated into practical treatment attempts for PD. GBA1 is another risk factor for PD, associated with damaged glucocerebrosidase function (Parlar et al., 2023). Ambroxol and a glucosylceramide synthase inhibitor (GZ667161) have been used to rescue damaged glucocerebrosidase activity and have proven effective in MOs (Frattini et al., 2025). Using clinically compliant differentiation protocols has enabled the derivation of multipotent neural progenitors with translational utility for cell replacement strategies addressing neurodegeneration in PD. Additionally, since organoids have a mixed differentiation characteristic, they exhibit a phenotype of dopaminergic neurons alongside astrocytes after differentiation, which is similar to the physiological situation and could potentially yield better therapeutic benefits (Kim et al., 2021). Researchers suggest that astrocytes derived from NSCs harvested from MOs are more therapeutic than those directly differentiated from iPSCs. A recent RNA sequencing study demonstrated that these organoids, after culture, showed increased expression of genes involved in phagocytosis, thereby enhancing the astrocytes’ ability to combat oxidative stress. Following the administration of these organoids into PD models, a greater amelioration of dopamine neuron loss was noted (Nam et al., 2024). Some researchers have attempted to use MOs for transplantation to treat motor deficits. MOs cultured for 15 days, according to their experimental protocol, were considered optimal for transplantation due to their high survival rate and cell maturation. Notably, after transplantation into the striatum of PD mice, significant improvements in motor capacity were recorded (Zheng et al., 2023). Such technologies could also be introduced into clinical practice, potentially curing movement impairments in patients. Additionally, extracellular vesicles obtained from organoids (OExo) through sequential centrifugation have been shown to effectively reduce oxidative stress. Furthermore, OExo are more effective than control cells from mesenchymal stem cells in stimulating the differentiation of dopaminergic neurons, making them a promising option for PD treatment (Ji et al., 2023).

### Huntington’s disease

HD is an autosomal-dominant neurodegenerative disorder caused by pathogenic CAG trinucleotide repeat expansions at the huntingtin locus, which express polyglutamine-expanded mutant huntingtin. This mutation leads to misfolding through conformational changes that result in proteotoxic nuclear inclusions (Handsaker et al., 2025). These clusters disrupt axonal transport and impair mitochondrial bioenergetics, ultimately causing selective striatocortical neurodegeneration (Berth and Lloyd, 2023; Tassone et al., 2023; Han et al., 2024). The monogenic pathogenesis of this disease manifests as a multidomain clinical illness characterized by hyperkinetic movement disorders, including choreoathetosis and dystonic posturing. Additionally, frontostriatal-mediated cognitive decline presents as working memory fragmentation and disintegration of attentional control, alongside neuropsychiatric comorbidities such as affective dysregulation syndromes and impulse control pathology. Together, these features describe the heterogeneous phenotypic spectrum of HD disease progression (Shafie et al., 2024).

#### Disease modeling

While organoid modeling of HD lagged behind its application in AD and PD research, recent breakthroughs demonstrate its growing potential for studying HD pathogenesis (Conforti et al., 2018; Miura et al., 2020; Lisowski et al., 2024). In 2018, researchers conducted a study utilizing HD- iPSCs. The findings indicated that HD cell lines containing excessive CAG repeat sequences exhibited defects in neuroectodermal development. These neural progenitor cells faced challenges in acquiring ventral telencephalon and striatal identities, as well as in neuronal maturation. Additionally, cortical neuron differentiation and cellular layering were adversely affected. With the advancement of organoid technology, researchers successfully generated HD COs, thus validating the conclusions drawn from iPSCs through the analysis of the abnormal transcriptome associated with these organoids (Conforti et al., 2018). In 2020, upon examining the transcriptome dataset, researchers discovered that retinoid X receptor gamma might be a key gene, as it ranked highly among the differentially expressed genes. Subsequently, SR11237, a selective agonist of retinoid X receptors, was added to the culture medium to facilitate the differentiation of lateral ganglionic eminence (LGE) cells. These differentiated LGE cells were then further induced to form HD hStrS, which can be used in HD investigations (Miura et al., 2020).

Even though organoids have not been extensively employed in the research of HD thus far, it is evident that researchers are introducing innovations in fundamental pathological models (**Additional Table 4**). For example, some researchers developed the hStrS into an organization of defined striosomal and matrix-like regions. They then created assembloids with COs, MOs, and hStrS to explore the detailed projections of neurons in the brain. Interestingly, they highlighted a breakthrough in their findings, including the discovery that COs project nerve fibers to hStrS, mirroring the *in vivo* pattern where early cortical innervation aligns with the sites of the striatum (Chen et al., 2022). The generation of striato-nigral assembloids represents a novel approach. Initially, neuroepithelial cells were treated with 20 ng/mL of sonic hedgehog to facilitate the induction of LGE cells, which were then further induced into striatal neurons. Finally, the striatal cell bodies were embedded onto an ultra-low-adherent 96-well plate near midbrain substantia nigra organoids for co-culture. To ensure that these assembloids were constructed properly and that all synapses formed between the components of the assembloids functioned correctly, immunofluorescence and optogenetic patch-clamp recording analyses were employed to obtain indirect evidence for the proper construction of the structure and the functionality of all inter-synaptic contacts in the striato-nigral assembloids (Wu et al., 2024a). The cell-cell interactions remain somewhat unclear, although scientists have attempted to uncover them and reveal their specific role in HD. To achieve this, mosaic telencephalic organoids were built. By fusing normal cells and HD cells for cultivation, this special type of organoid allows healthy cells to come into contact with HD cells within the same 3D structural system (Galimberti et al., 2024).

**Additional Table 4:** Promotion of organoids in HD and ALS

Disease	Promotion	Approaches	Reference
Huntington's disease	Assembloids construction	First developed human hStrS with striosome and matrix-like compartments. Then assembled the assembloids by combining hStrS with COs and MOs.	Chen et al., 2022
		Initially treated neuroepithelial cells with 20 ng/mL of sonic hedgehog to promote the induction of LGE cells. Differentiated them into striatal cells subsequently. The generated striatal components were placed in an ultralow-attachment 96-well plate in close proximity to SN organoids, enabling their fusion.	Wu et al., 2024a
Amyotrophic lateral sclerosis	Matrigel replacement	Replaced Matrigel with alginate hydrogels to produce spinal cord organoids.	Chooi et al., 2023

ALS: Amyotrophic lateral sclerosis; COs: cerebral organoids; hStrS: human striatal spheroids; HD: Huntington's disease; LGE: lateral ganglionic eminence; MOs: midbrain organoids; SN: substantia nigra.

#### Target discovery and therapeutic strategies of Huntington’s disease

The pathological damage of mutant huntingtin (mHTT) to brain development is evident in striatal organoids, which demonstrate a strong connection between HD and mitochondrial dysfunction. Mitochondrial heat shock factor 1 (mtHSF1) initiates mitochondrial fission by activating the phosphorylation of dynamin-related protein 1 (Drp1) and inhibiting the oligomerization of single-stranded DNA-binding protein 1 (SSBP1). This suppression is critical because it leads to the subsequent deletion of mitochondrial DNA, contributing to neurodegeneration. Having successfully rescued pathological phenotypes in HD striatal organoids, DH1—a peptide inhibitor that prevents HSF1 from co-localizing with mitochondria—has been regarded as a therapeutic possibility for HD (Liu et al., 2022). Coiled-coil-helix-coiled-coil-helix domain-containing protein 2 (CHCHD2) has emerged as an essential molecule involved in mitochondrial respiratory chain activity, mitochondrial homeostasis, and mitochondrial autophagy, all of which are critical for the normal maintenance of mitochondrial morphology and function (Ren et al., 2024b). In HD organoids, the level of CHCHD2 decreases, leading to abnormal mitochondrial morphology and dynamics, and resulting in an imbalanced mitochondrial integrated stress response (mISR). This finding suggests that CHCHD2 holds great potential for the treatment of HD (Lisowski et al., 2024).

Efforts to apply organoid-based therapies are also underway. To develop mosaic telencephalic organoids containing both healthy and HD cellular populations, investigators co-cultured HD iPSCs with healthy control iPSCs. Thorough characterization of the mosaic organoids indicated that healthy cells exert a strong non-cell-autonomous effect on HD cells through cell-to-cell contact. Specifically, these intercellular interactions can restore the identity and gene expression profiles of the HD cells and rectify their developmental program, steering it back toward canonical neurodevelopment. This contributes to an increase in synaptic density as well as connectivity with the surrounding environment (Galimberti et al., 2024).

### Amyotrophic lateral sclerosis

ALS is a NDD that progressively leads to the selective degeneration of upper motor neurons in the corticospinal tract and bulbar or spinal lower motor neurons, resulting in skeletal muscle denervation and the disintegration of neuromuscular junctions (Rizea et al., 2024). Clinically, ALS presents with signs of lower motor neuron damage, such as fasciculations and hyporeflexia, as well as signs of upper motor neuron damage, including spasticity and hyperreflexia. Progressively, there is a manifestation of bulbar damage, characterized by dysarthria and dysphagia. Ultimately, the deterioration causes respiratory failure due to paralysis of the diaphragm (Rizea et al., 2024). Although the pathological mechanism driving ALS remains unknown, several contributing factors have been suggested, including mitochondrial defects, excitotoxicity, and neuroinflammation (Van Daele et al., 2024).

#### Disease modeling

Compared to various NDDs discussed earlier, ALS exerts particularly severe damage on the motor system. The investigation of ALS using organoids began with a focus on motor-related tissues. In patients with sarcoma (FUS) mutations associated with ALS, the fused in FUS protein, which is normally localized within the nucleus, can mislocalize to the cytoplasm, leading to toxic effects on motor neurons (Kour et al., 2023). In 2019, a study employed iPSC technology combined with a microfluidic system to explore the impact of FUS mutations on axon branching in motor neurons (MNs) affected by ALS. By comparing isogenic iPSC lines—one harboring the FUS mutation and one without—the researchers observed that MNs carrying the FUS mutation exhibited aberrant axon branching correlated with elevated Fos-B mRNA levels. To enhance differentiation between axons and dendrites, motor neuron precursor cells (MPCs) were not fully dissociated into single cells for culturing; instead, they were maintained in spherical forms within a microfluidic device. This device comprised two chambers linked by a central channel, where organoids were placed in one chamber while their extended axons traversed the connecting channel. Researchers could directly sever this channel to collect axons for subsequent RNA analysis. Alterations in neuromuscular organoids reflected damaged axons, and transcriptome analysis validated the critical role of Fos-B in these processes (Akiyama et al., 2019). The spinal cord’s role in ALS pathogenesis is underscored by its susceptibility to motor neuron degeneration and protein aggregation pathology, highlighting the need to build spinal cord organoids (SCOs) (Ogura et al., 2018; Alarcan et al., 2022). In previous studies, SCOs were constructed using a suspension culture system (Ogura et al., 2018). This method enabled the development of dorsal spinal cord organoids made from hPSCs, resulting in self-organization into dorsal spinal cord patterns containing roof-plate-like dorsal niches that secrete BMP/WNT morphogens, essential for patterning dorsal progenitor domains. Later ventralization, achieved by perturbing the SHH gradient, yields intermediate spinal and ventral domains (Ogura et al., 2018). Optimization of SCOs has been placed on the agenda, with a focus on their specificity (**Additional Table 4**). For instance, researchers have applied alginate hydrogels as a substitute for Matrigel in the production of SCOs. They reported that this method not only demonstrates similar efficiency and speed but also improves representativeness. The scientists suggested that many of the proteins expressed in Matrigel are unnecessary for the formation of spinal cord organoids. In fact, when culturing SCOs in Matrigel, they found that bioactive factors negatively impact this process; the presence of these factors leads to stronger expression of brain markers FOXA2 and forkhead box G1 (FOXG1), resulting in a loss of spinal cord regionalization in spinal organoids (Chooi et al., 2023).

#### Target discovery and therapeutic strategies of amyotrophic lateral sclerosis

Through the use of COs and spinal cord organoids, researchers mechanistically queried the network of FUS-mediated regulation. They found experimental evidence that FUS makes stereospecific binding to a neurotrophic receptor tyrosine kinase 3 (Ntrk3) mRNA transcript. In spinal cord organoids, the increase in the truncated non-catalytic form (TR-Ntrk3) was greater than that of the full-length kinase-active form (FL-Ntrk3), impairing self-renewal and the generation of progenitor cells/motor neurons in the spinal cord organoids. Most critically, knockdown of Ntrk3 using shRNA successfully rescued the developmental defects caused by FUS loss in spinal cord organoids, providing robust evidence of pathway epistasis (Zou et al., 2022). Dysfunction of the neuromuscular junction (NMJ) occurs early in the disease course of ALS, long before the death of motor neurons. This suggests that NMJ damage may be one of the early events in the onset of ALS (Sun et al., 2024). Mutations in C9orf72 also represent significant factors contributing to disease pathology. Neuromuscular organoids derived from iPSCs of C9orf72-ALS patients displayed NMJ dysfunction characterized by reduced contractility of skeletal muscle due to impaired innervation and subsequent Schwann cell death. In the context of C9orf72-ALS, mutations result in loss-of-function effects on the C9orf72 protein—an essential regulator of autophagy—leading to elevated levels of autophagy and the accumulation of toxic dipeptide repeat proteins (DPRs) (Gao et al., 2024). Neuroinflammatory factors play a crucial role in the pathology of ALS, and organoids have been employed in related research efforts. Spinal cord organoids harboring C9orf72 gene mutations exhibit an upregulation of pro-inflammatory factors, including activation of the P53 signaling pathway, WNT signaling pathway, and TGF-β signaling pathway (Guo et al., 2024).

Stress granules (SGs) are membrane-less RNA aggregates that form in the cytoplasm of eukaryotic cells through liquid-liquid phase separation under various stress conditions. The core SG population contains abundant untranslated mRNA and multiple RNA-binding proteins (RBPs). SGs function as cellular mechanisms to buffer against multiple types of environmental stress (Yang et al., 2023). COs are subjected to energy deficiency, where energy deficits induce the formation of SGs through the mTOR-4EBP1 pathway; however, they prevent the clearance of SGs by impairing the RNA-binding activity of eukaryotic initiation factor 4A-I (eIF4A1), resulting in smaller SGs with reduced degradation kinetics (Wang et al., 2022). ATAXIN-2 (ATXN2) has been found to play diverse roles in cellular physiological processes such as stress response, cell growth, and metabolism. An expanded polyglutamine repeat at the N-terminal of ATXN2 is linked to a moderate increase in ALS risk. In NM organoids, researchers observed impaired respiratory mitochondrial function and increased inflammation. This may occur because ATXN2 is an RNA-binding protein involved in SG assembly; therefore, a mutation in this protein alters SG formation, resulting in a defective cellular stress response and neuronal apoptosis (Vieira de Sá et al., 2024).

Validation of potential therapeutic approaches is conducted in organoids. Recent work utilized SB431542 and dorsomorphin to induce neuronal fate, resulting in rosebud-forming neurons and microglial progenitor cells. This strategy was employed to develop brain organoids containing microglia. Compared with the control group, the expression of genes related to the type I interferon signaling pathway was significantly reduced in the ALS organoids. At the same time, exogenous IFN-γ therapy caused an increase in interferon-stimulated effector mediators, induced an M2-like reparative phenotype of microglia, and enabled phagocytosis (Hong et al., 2023).

### Frontotemporal dementia

FTD is a heterogeneous group of neurodegenerative disorders characterized by progressive behavioral, language, and motor impairments due to the degeneration of the frontal and temporal lobes (Clayton et al., 2024). Pathologically, FTD is marked by the accumulation of abnormal proteins, such as TDP-43 (Casiraghi et al., 2025), dipeptides (Li et al., 2023), and tau (Chen et al., 2023), in neurons and glial cells, leading to neurodegeneration and gliosis (Veverová et al., 2025).

#### Disease modeling

A study in 2017 explored how cyclin-dependent kinase 5 (Cdk5), an enzyme that modifies tau proteins, interacts with P25 to drive the development of FTD through abnormal protein interactions (Seo et al., 2017). Similar to some researchers in Alzheimer’s disease (AD) research (Holubiec et al., 2023; Kim et al., 2024b), the researchers constructed organoids using fibroblasts from FTD patients. Following the successful establishment of tauopathy-associated cortical organoids, comparative proteomic analysis revealed that mutant models exhibited elevated p25/p35 proteolytic cleavage ratios relative to isogenic controls (Seo et al., 2017). Since then, organoids have been widely used in FTD-related research. Two years later, another type of cortical organoid characterized by a mutation in the microtubule-associated protein tau (MAPT) gene was constructed, featuring a R406W tau mutation. This missense mutation causes patients to exhibit ALS-like symptoms but not typical FTD features (Nakamura et al., 2019).

#### Target discovery and therapeutic strategies of frontotemporal dementia

Organoids can be cultured over extended periods, and interventions during the cultivation process are highly feasible. This enables researchers to conduct long-term studies using organoid models (Hendriks et al., 2024; Lorenzo-Martín et al., 2025). Mutations in the MAPT gene lead to the pathological accumulation of tau and the death of glutamatergic cortical neurons. However, the process underlying this pathological condition is not well understood in terms of the specific pathological features at each stage. To gain a clearer understanding, researchers constructed tau-V337M mutant brain organoids for long-term observation. At two months, the organoids showed signs of stress response and excitotoxicity, with increased expression of MAPT, glutamatergic signaling pathways, and regulatory factors such as the RNA-binding protein ELAV-like RNA binding protein 4 (ELAVL4), along with an increase in stress granules. Over the next four months, changes including splicing alterations, disrupted autophagy, and the accumulation of tau and phosphorylated tau gradually occurred. By six months, the specific loss of glutamatergic neurons, a characteristic of FTD as a NDD, became evident (Bowles et al., 2021).

Another study focused on the relationship between the progranulin gene (GRN) and TDP-43. Furthermore, they utilized a novel protocol to create 3D models. Instead of initializing the organoids from iPSCs, the cells were first derived into a primary stage as cortical-like neurons and cortical-like astrocytes. These were then mixed in a designed pattern of numbers and ratios to produce mature brain organoids (mbOrgs). In GRN-deficient mbOrgs, FTD-related pathological features, including TDP-43 mislocalization, hyperphosphorylation, and loss-of-function (LoF) phenotypes, emerged spontaneously. Dysfunctional astrocytes in mbOrgs were considered the culprits, exhibiting reduced expression of phagocytosis-related genes, including MERTK (MER proto-oncogene, tyrosine kinase) and MEGF10 (epidermal growth factor-like domains 10). Moreover, mbOrgs composed of GRN-deficient astrocytes and normal neurons still exhibited the typical FTD phenotype, providing new evidence that non-neuronal cells drive neuronal death (de Majo et al., 2023).

## Current Status of Clinical Translation and Application

As the potential of organoid technology continues to be explored, an increasing number of funding projects worldwide are emerging regarding its application in the research and technological innovation of NDDs. We have organized these projects and plans and provided a brief overview (**Box 1**).

Box 1: Registered clinical trials and funded projects

**1. Developing new therapeutic strategies to counteract synaptic and cognitive deficits in Alzheimer's disease by targeting zDHHC enzymes (Registered Clinical Trials)**

**Clinical Trial Identifier:** NCT06677632, 2024, Italy

**Design Features:** Intervention of 2-BP to inhibit S-palmitoylation targeting zDHHC S-acyltransferases to treat counteract synaptic and cognitive deficits.

**Practical Significance:** This approach can lead to novel therapies that modify protein function in AD by targeting specific enzymatic pathways involved in synaptic dysfunction, potentially slowing disease progression and improving cognitive outcomes.

**2. Mesenchymal stem cell secretome for neuroinflammation (Registered Clinical Trials)**

**Clinical Trial Identifier:** NCT06551649, 2024, Italy

**Design Features:** Use the secretome of hAMSC to alleviate chronic inflammation in NDDs.

**Practical Significance:** This study can lead to an innovative therapeutic strategy that not only effectively counteracts neurodegeneration but also reduces inflammation.

**3. A blood-to-neural lineage reprogramming platform for personalized cell and brain organoid models to advance drug discovery for rare neurological disorders (Registered Clinical Trials)**

**Clinical Trial Identifier:** ChiCTR2500099973, 2025, China

**Design Features:** Establish stable cell lines of iPSCs derived from ALS patients through direct neurogenic lineage reprogramming methods in ALS patient iPSC-derived organoids.

**Practical Significance:** This platform enables personalized disease modeling and high-throughput drug screening for ALS, facilitating precision medicine and accelerating therapeutic development for rare neurological conditions.

**4. Elucidating immune and microglial dynamics in Alzheimer's disease with advanced organoid models. (Projects funded by NIH)**

**Reference:** Burgess, 2025, the United States

**Design Features:** Advanced organoid models incorporating immune and microglial components to study neuroinflammation dynamics.

**Practical Significance:** By incorporating immune components, these models provide critical insights into neuroimmune interactions, aiding the development of immunomodulatory treatments and biomarkers for AD and related disorders.

**5. Perfusable brain organoids: a long-term culture approach (projects funded by NIH)**

**Reference:** Coppola, 2020, the United States

**Design Features:** Development of perfusable brain organoids maintained through transplantation in murine hosts for long-term survival.

**Practical Significance:** Creates more physiologically relevant models with vascular components, enhancing organoid maturation and experimental applications.

**6. 3D *in vitro* model of genetic and sporadic FTD: accelerating novel therapeutic development (projects funded by NIH)**

**Reference:** Ghaffari, 2025, the United States

**Design Features:** Create organoids for FTD and ALS research.

**Practical Significance:** These models accelerate drug discovery by providing disease-relevant platforms for testing therapeutics targeting FTD and ALS pathologies, reducing reliance on animal models and streamlining preclinical research.

**7. ADRD induced pluripotent stem cell/organoid core (projects funded by NIH)**

**Reference:** Ellerby, 2023, the United States

**Design Features:** Research on aging microglia integration in organoids.

**Practical Significance:** Integrating aged microglia improves the relevance of organoid models for age-related NDDs, enabling better study of neuroinflammation in aging and identifying age-specific therapeutic targets.

**8. Treatment of short-term memory deficits in AD mice using precisely controllable cortical organoids (Projects funded by NSFC)**

**Reference:** Chaojuan, 2021, China

**Design Features:** Treat short-term memory deficits of AD mice with cortical organoids

**Practical Significance:** This demonstrates the therapeutic potential of organoid-based interventions for cognitive symptoms, paving the way for regenerative approaches and cell replacement therapies in AD treatment.

**9. Mechanism of amyloid-β accumulation and apoptosis induced by BACE2 deficiency using brain organoids differentiated from human-induced pluripotent stem cells (projects funded by NSFC)**

**Reference:** Peng, 2021, China

**Design Features:** Study the mechanism of β-amyloid accumulation and apoptosis caused by BACE2 deficiency based on brain organoids differentiated from human-iPSCs

**Practical Significance:** This research identifies new pathways in amyloid pathology, revealing potential therapeutic targets for reducing amyloid burden and neuronal apoptosis in AD, which could inform future drug design.

**10. Cerebral organoids: human mini brains in a dish open up new possibilities for drug development in neurodegenerative and developmental diseases (projects funded by the European Research Council)**

**Reference:** Knoblich, 2017, Austria

**Design Features:** Use cerebral organoids as a platform for drug development in NDDs

**Practical Significance:** These organoids serve as high-throughput screening tools for drug discovery, accelerating the development of treatments for NDDs by providing a human-relevant platform that reduces ethical and practical limitations of animal models.

**11. Generation of position specific organoids to study human neuromuscular system development and disease (projects funded by the European Research Council)**

**Reference:** Gkouti, 2022, Germany

**Design Features:** Create position-specific neuromuscular organoids

**Practical Significance:** These organoids enable precise modeling of neuromuscular disorders, facilitating the study of region-specific pathologies and the development of targeted therapies for conditions such as muscular dystrophy and motor neuron diseases.

Globally, various discoveries related to neurodegenerative diseases based on organoid technology have gradually been translated into clinical applications. Several projects utilizing organoids have been registered on the North American Clinical Trials Registry. In Italy, organoids derived from iPSCs of patients with AD revealed an increase in S-palmitoylation of AD-related proteins, such as APP and beta-secretase 1. This supports the idea that intervention with 2-bromopalmitate (2-BP), which inhibits S-palmitoylation by targeting zinc finger DHHC domain-containing (zDHHC) S-acyltransferases, could be effective in treating AD-related synaptic and cognitive deficits (NCT06677632, 2024). Also in Italy, there is a trial using the secretome of human amniotic mesenchymal stromal cells to alleviate chronic inflammation in NDDs. One of the researchers’ goals in this trial is to develop customized organoid models for ALS patients as an accurate platform for validating therapeutic strategies (NCT06551649, 2024). In China, a study aiming to establish stable cell lines of iPSCs derived from ALS patients through direct neurogenic lineage reprogramming methods in ALS patient iPSC-derived organoids was registered in Hong Kong by scholars (ChiCTR2500099973, 2025).

Sufficient financial resources are necessary for developing scientific research. We are delighted to see that in most parts of the world, many projects on organoids and NDDs are being funded. In the U.S., several projects have received support from the National Institutes of Health (NIH). Scientists at the University of North Carolina at Chapel Hill were funded for their work analyzing immune and microglial dynamics in AD and PD, with a particular emphasis on employing sophisticated organoid models (Burgess, 2025). Researchers at Yale University received funding for developing a type of perfusable brain organoid that can survive long-term by being transplanted into mice (Coppola, 2020). A program aimed at creating organoids for FTD and ALS research was also funded (Ghaffari, 2025). Researchers at the Buck Institute were funded for their research on aging microglia integration in organoids (Ellerby, 2023). Numerous projects are being funded in China as well. For example, Researchers at Sun Yat-sen University received funding to study the mechanism of Aβ accumulation and apoptosis caused by BACE2 deficiency, using brain organoids differentiated from human iPSCs (Peng, 2021). In Austria, researchers funded by the European Research Council (ERC) utilized COs as a platform for drug development in NDDs (Knoblich, 2017). In Germany, researchers were also funded by the ERC to create position-specific neuromuscular organoids (Gkouti, 2022). In clinical studies on NDDs, organoids have primarily been implemented as complementary pathological models, primarily serving to explore disease mechanisms and facilitate drug screening, as organoids can recapitulate the hallmarks of neurons or synapses with spatial resolution. However, to date, very few clinical trials and FDA-approved therapeutic applications utilize organoid-based products. This suggests that, despite the growing interest in organoids for clinical applications and the increasing reliance on organoid technology in trials, the number of therapeutic applications based on organoids remains limited. The promise of organoids as therapeutic products to support clinical trials has not yet been fulfilled; the potential of organoids for clinical use has yet to be fully realized. As we described previously (Ji et al., 2023), some organoid-derived outputs may potentially be used directly in clinical settings. We encourage future work to focus more on these applications to help advance organoid technology in the translational development process from the laboratory bench to the clinical bench.

## Limitations

This review has several limitations. To incorporate more notable insights into our article, we did not impose a strict quality control barrier, which may result in the inclusion of research with less potential for clinical application. Our review focuses primarily on the preclinical research of organoid technology in NDDs. Most investigations involving organoids in NDDs remain at the laboratory research stage, utilizing organoids as platforms to identify targets and validate drugs. The application of organoids in clinical practice requires ongoing attention and further exploration by researchers.

## Conclusions

This review explores the research methods of organoids, identifies their inherent limitations, and introduces the application and development of organoids in specific NDDs. As organoid technology has become a focal point for researchers studying NDDs, many reviews of relevant investigations have emerged in recent years (Jusop et al., 2023; Lei et al., 2024). The application of organoids in NDDs has been summarized by several authors (Jusop et al., 2023; Lei et al., 2024), and many innovations in organoid technology have also been well documented (Ouaidat et al., 2025; Pagliaro et al., 2025; Sun et al., 2025). However, few reviews have combined these aspects to provide specific research paradigms. We hope our work can supplement current reviews by integrating the application and optimization of organoids in NDDs. Through the paradigms presented in this article regarding the application of organoids derived from technological innovations in NDDs, we aim to deepen researchers’ understanding of this model, assist them in optimizing it, and facilitate the entire research process in NDDs—from disease modeling to target identification and the formulation of treatment strategies—ultimately enhancing our ability to study NDDs.

Organoid technology can be considered a groundbreaking advancement in NDD research, as it enables the most physiologically accurate modeling of human-specific neurodevelopmental processes and NDD mechanisms within a biologically relevant *in vitro* 3D environment (Pagliaro et al., 2025). By recapitulating the spatial complexity and cellular interactions of the human brain, organoids bridge the gap between conventional 2D cultures and animal models, allowing researchers to dissect disease mechanisms, validate therapeutic targets, and screen for potential interventions with markedly improved fidelity. With the advent of culturing technologies, researchers can enhance organoid production fidelity by fine-tuning developmental processes and harnessing genetic engineering strategies (Qian et al., 2018; Zhao et al., 2020). Microfluidic-based platforms facilitate strict spatiotemporal tuning of the culture microenvironment, improving organoid maturation consistency and experimental repeatability (Liu et al., 2024a). Bioprinting allows for the manufacturing of custom-constructed organoids with specific cellular compositions and 3D architectures (Juraski et al., 2023). Grafts and assembloids promote the engorgement of engineered organoids to mitigate design challenges such as death-ring development (Wang et al., 2023; Onesto et al., 2024). These innovations have deepened our understanding of neurodegenerative processes, from Aβ and tau pathology in AD (Liu et al., 2024b) to alpha-synuclein aggregation in PD (Kim et al., 2023), while also revealing novel insights into mitochondrial dysfunction, neuroinflammation, and synaptic toxicity (Bowles et al., 2021; Liu et al., 2022; Mi et al., 2023).

In the future, as organoid technology integrates with multi-omics profiling (You et al., 2024) and artificial intelligence (Renner et al., 2021), patient-derived iPSCs are likely to accelerate personalized medicine approaches (Zhao et al., 2020), enabling the development of tailored therapies for genetically diverse patient populations. Additionally, the capacity of organoids to model pathogen interactions (Kong et al., 2023) and the effects of environmental toxins (Lu et al., 2024) positions them as indispensable tools for unraveling the multifactorial etiology of neurodegenerative disorders.

## Additional files:

***Additional Table 1:*** *Primary searching results.*

***Additional Table 2:*** *Promotion of organoids in AD.*

***Additional Table 3:*** *Promotion of organoids in Parkinson’s disease.*

***Additional Table 4:*** *Promotion of organoids in HD and ALS.*

## Data Availability

*All relevant data are within the paper and its Additional files.*
